# Trigeminal neuralgia treatment outcomes following Gamma Knife radiosurgery with a minimum 3-year follow-up

**DOI:** 10.1007/s13566-013-0134-3

**Published:** 2013-11-20

**Authors:** Sana D. Karam, Alexander Tai, Margaux Wooster, Abdul Rashid, Rosanna Chen, Nimrah Baig, Ann Jay, K. William Harter, Pamela Randolph-Jackson, Adedamola Omogbehin, Edward F. Aulisi, Jeff Jacobson

**Affiliations:** 1 Department of Radiation Oncology, Medstar Georgetown University Hospital, 3800 Reservoir Rd., NW, Washington, DC 20007 USA; 2 Department of Radiation Oncology, Medstar Washington Hospital Center, Washington, DC USA; 3 Department of Radiology, Medstar Georgetown University Hospital, Washington, DC USA; 4 Department of Neurosurgery, Medstar Washington Hospital Center, Washington, DC USA

**Keywords:** Trigeminal neuralgia, Tic doloreaux, Radiosurgery, Gamma Knife, Long term

## Abstract

**Objective:**

Effective short-term outcomes have been well documented for trigeminal neuralgia (TN) patients treated with Gamma Knife radiosurgery (GKRS) with reported success rates of 70–90 % with median follow-up intervals of 19–75 months. Fewer series, however, have described uniform long-term follow-up data. In this study, we report our long-term institutional outcomes in patients treated with GKRS after a minimum follow-up of 36 months.

**Methods:**

Thirty-six consecutive patients with medically intractable TN received a median radiation dose of 45 Gy applied with a single 4-mm isocenter to the affected trigeminal nerve. Follow-up data were obtained by clinical examination and telephone questionnaire. Outcome results were categorized based on the Barrow Neurological Institute (BNI) pain scale with BNI I–III considered to be good outcomes and BNI IV–V considered as treatment failure. BNI facial numbness score was used to assess treatment complications.

**Results:**

The incidence of early pain relief was high (80.5 %) and relief was noted in an average of 1.6 months after treatment. At minimum follow-up of 3 years, 67 % were pain free (BNI I) and 75 % had good treatment outcome. At a mean last follow-up of 69 months, 32 % were free from any pain and 63 % were free from severe pain. Bothersome posttreatment facial numbness was reported in 11 % of the patients. A statistically significant correlation was found between age and recurrence of any pain with age >70 predicting a more favorable outcome after radiosurgery.

**Conclusion:**

The success rate of GKRS for treatment of medically intractable TN declines over time with 32 % reporting ideal outcome and 63 % reporting good outcome. Patients older than age 70 are good candidates for radiosurgery. This data should help in setting realistic expectations for weighing the various available treatment options.

## Introduction

Trigeminal neuralgia (TN), also known as tic doloreaux, is a debilitating pain condition characterized by agonizing, paroxysmal, and lancinating pain [1]. First-line treatment includes anticonvulsant and antidepressant, but this often fails to provide pain relief and is associated with various side effects, leading patients to seek other treatments options [1]. Second-line treatment modalities are given to patients whose symptoms are intractable or who cannot tolerate medication. These include surgical procedures such as microvascular decompression, and ablative procedures such as percutaneous balloon microcompression, radiofrequency rhizotomy, glycerol rhizolysis, and Gamma Knife radiosurgery (GKRS). The short-term outcomes of patients treated with GKRS are well described [2]. Initial publications with the GKRS system at short median follow-up of approximately 2 years report pain relief outcomes in 70–90 % of their patients and pain-free status in 20–60 % [3–7]. As longer series with median follow-up of 19–75 months started to emerge, pain relief rates ranging from 65 to 85 % were reported [5, 8–10]. However, failure to exclude patients with short-term follow-up in long-term analysis muddied the interpretation and prevented a clear understanding of longevity of GKRS's effectiveness in controlling TN pain. More recently, a few studies have examined long-term follow-up of GKRS in controlling pain with variable outcomes [11–15]. Here, we present our long-term institutional outcomes of GK in the treatment of TN by restricting our analysis to include only those with a minimum follow-up of 36 and after only a single GK treatment.

## Methods

### Patient characteristics

After institutional review board approval, patient demographic characteristics, clinical presentation, treatment history, and the radiosurgical modality were retrospectively reviewed.

Patients were also followed up by a telephone questionnaire that was conducted by a medical resident and a medical student who were not involved in treatment. Patients were questioned about the time to the onset of pain relief, the degree of pain relief, and treatment complications. Based on the Barrow Neurological Institute (BNI) score for TN, we classified pain relief after treatment into five grades. A BNI I score corresponded to complete pain relief without medications; BNI II score, some pain but not requiring medications; BNI III score, some pain but adequately controlled with medications; BNI IV score, some pain not adequately controlled with medication; and BNI V score, severe pain or no pain relief. The BNI facial numbness score was used to assess complications. A BNI I score corresponded to no facial numbness; BNI II score, mild facial numbness, not bothersome; BNI III, facial numbness somewhat bothersome; and BNI IV score, facial numbness, very bothersome.

Between September 2002 and June 2010, 81 patients with TN underwent GKRS at our clinic. Indications for GKRS included medically intractable pain refractory to standard medications, refusal of invasive procedures, or failure of previous invasive procedures. Among them, we retrospectively reviewed the medical records of patients with TN undergoing GKRS treatment that had a minimum follow-up of 36 months. Ten patients had died prior to their 36 months follow-up. Fourteen patients were lost to follow-up and were lost to follow-up and on whom we were unable to obtain accurate contact information. Five patients had received GKRS treatment twice and were excluded. Two patients had bilateral disease and were excluded. Eleven patients refused to participate in the survey and adequate follow-up could not be obtained on the remaining 20 patients despite valid phone numbers and multiple attempts. Three patients were retreated but results of GKS retreatment were not included in this analysis. Rather, our report presents the results of single GKS treatment for patients with TN when using a standard treatment algorithm. A total of 36 patients were included in the final analysis with a mean follow-up of 69 months (range 36–246 months). A summary of patient characteristics is provided in Table 1. None of the patients included in the final analysis had multiple sclerosis.

**Table 1:** Clinical demographic characteristics in 36 patients with medically intractable trigeminal neuralgia treated with Gamma Knife radiosurgery (GKRS)

Characteristic	Value
Gender	
Male	14 (39 %)
Female	22 (61 %)
Mean age (range)	71 (29–97)
Prior surgery	4 (11 %)
Median duration in years (range)	6 (0.25–30)
Pain distribution	
V2	17 (47 %)
V3	11 (30.5 %)
V2,3	6 (17 %)
V1,2,3	2 (5.5 %)
Side of pain	
Right	15 (42 %)
Left	21 (58 %)

### Radiosurgery technique

All patients were treated on the 201-source 60Co Gamma Knife unit, model 4C, manufactured by Elekta instruments (Elekta, Norcross, Georgia). Treatment planning was performed jointly by a radiation oncologist, neurosurgeon, and medical physicist for all cases. After induction of local anesthesia, the Leksell Model G stereotactic coordinate frame was affixed to the head of each patient, and contrast enhanced MR imaging was performed to visualize and target the trigeminal nerve root entry zone. As a planning tool, the Leksell Gamma Plan System (Elekta) was used. A single 4-mm isocenter was placed adjacent to the trigeminal nerve root entry zone. The median peripheral dose was 45 Gy with a range of maximum dose between 80 and 90 Gy delivered to the involved trigeminal nerve root entry zone. A plugging pattern typically blocking 32 sources was used so that the surface of the brainstem was irradiated at no greater than the 20 % isodose line for any patient.

### Statistical analysis

Treatment outcomes were assessed by patient self-reports of pain control and medication usage at last follow-up. A pain-free outcome was defined as BNI pain score I and pain relief or good outcome was regarded as maintaining a BNI pain score IIIa or better without requiring further surgery. Treatment failure was defined as pain returning to a BNI level of IV or V, or the patient undergoing an invasive surgical procedure due to uncontrolled pain. A recurrence was defined as a relapse to a previous lower level after attainment of any higher level of pain relief. Patients reported the time interval for a response and pain recurrence after GKRS. The date of treatment failure was considered to be the date at which pain relief became a BNI IV or V score.

Time to BNI class IV to V pain relapse was calculated with the Kaplan–Meier method. Log rank tests were performed to determine statistical differences between pain relapse curves. We conducted a univariate analysis of several factors hypothesized to influence or predict successful treatment, using Cox regression analysis: age, gender, side of pain, duration of symptoms, new facial numbness. A *p* value <0.05 was accepted as statistically significant. All statistical calculations were performed using SPSS software, version 13.0 (SPSS, Inc., Chicago, IL, USA).

## Results

### Pain relief after GKRS

**Fig. 1:**
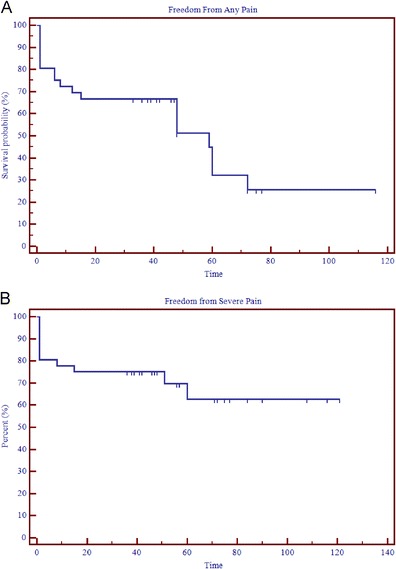
**a** Kaplan–Meier curve for time from Gamma Knife radiosurgery (GKRS) and freedom from any pain (BNI I). **b** Kaplan–Meier curve for time from Gamma Knife radiosurgery (GKRS) to Barrow Neurologic Institute (BNI) class IV to V pain relapse (i.e., freedom from severe pain)

The mean time from GKRS to pain improvement was 1.6 months (range, 1 day–6 months). Of those patient that experienced immediate pain relief (29 or 80.6 %), the majority (27 or 75 %) described an early pain response to treatment within the first 2 months after GKRS, whereas two (5.5 %) patients reported symptoms relief after 2 months. Seven patients (19 %) reported no pain response. The median time to recurrence of any symptoms was 46 months (range, 6–116 months). Of the 29 patients that experienced pain relief, 17 (58.6 %) did not have any pain recurrence (BNI I), while the remaining 12 (41.4 %) had some return of some pain (BNI ≥2). Results of Kaplan–Meier analysis of response time after GKRS are displayed in Fig. 1. At median follow-up of 5 years, actuarial freedom from any pain recurrence (BNI ≥2) is 40 %.

At last follow-up, 26 (72.2 %) patients had good treatment outcome (BNI I, II, or III) and 10 patients (27.8 %) had treatment failure. The 10 patients with treatment failure included the seven patients who did not have any response to treatment. Kaplan–Meier analysis shows an actuarial rate freedom from severe pain of 63 % at mean last follow-up of 69 months (Fig. 1b). The patient responses at last follow-up, as determined using the BNI pain intensity scoring system, are listed in Table 2. At last follow-up, 14 (39 %) of the patients had no pain and no medications (BNI I) with a median time of maintaining a BNI I pain score of 46 months (range, 36–77 months). A summary of pain outcomes at last follow-up is listed in Table 3.

**Table 2:** Pain response (top 5 rows) and development of facial numbness post treatment (bottom 3 rows) at last follow-up

	Number of patients (%)
BNI pain intensity scale	
BNI I	14 (39)
BNI II	3 (8)
BNI III	8 (22)
BNI IV	8 (22)
BNI V	3 (8)
BNI facial numbness score	
BNI I–II	32 (89)
BNI III	0 (0)
BNI IV	4 (11)

**Table 3:** Summary of actuarial rates of pain outcomes at last follow-up

	Actuarial rate at 36 months (%)	Actuarial rate at 69 months (%)
Freedom from any pain (*n* = 36)	67	32
Freedom from any pain recurrence in responders (*n* = 29)	83	40
Freedom from severe pain or good treatment outcome (*n* = 36)	75	63

### Treatment-related complications

The majority of the patients (26 or 72.2 %) did not experience any post-treatment facial numbness. Two patients (16.7 %) reported mild facial numbness that was not bothersome. Very bothersome facial numbness (BNI IV) was reported in a total of four patients (11.1 %) (Table 2). However, all four patients had good pain treatment outcome with three reporting a posttreatment BNI pain intensity score of I and one patient reporting a BNI pain score of II at longest follow-up. All four had received a treatment dose of 45 Gy. There were no reports of decreased corneal sensation (dry eye syndrome).

### Prognosticators

Univariate analysis for previously published prognosticators of treatment failure is shown in Table 4. No statistically significant correlation was found between treatment failure and age, gender, dose, laterality of pain, duration of symptoms, or development of new bothersome facial numbness. There was, however, a statistically significant difference between age and recurrence or persistence of any pain (BNI >1) and age (*p* = 0.02). Patients older than 70 reported significantly better freedom from any pain compared with those less than 70 years of age.

**Table 4:** Summary of various prognosticators and development of treatment failure (BNI IV–V)

Variable	Good outcome	Treatment failure	*p* value
No of patients	25 (69.4 %)	11 (30.6 %)	0.03
Gender			
Male	16	6	0.87
Female	9	5	
Age			
≥70 years	15	4	0.34
<70 years	10	7	
Side of pain			
Right	9	6	0.50
Left	16	5	
Duration of symptoms			
>18 months	5	5	0.20
<18 months	16	4	
Dose			
40 Gy	7	5	0.52
45 Gy	18	6	
New bothersome facial numbness	4	0	0.41

## Discussion

In their large series, Kondziolka et al. [2] and Régis et al. [7] reported that GKRS was a safe and effective alternative treatment to surgical procedures in the treatment of medically intractable TN. However, treatment outcomes and treatment-related complication rates have varied according to follow-up period, time for assessment of pain outcome, and different pain scores. In the present study, we report on pain outcome and side effects using standardized pain scores with a minimum 3-year follow-up to evaluate the long-term results of GKRS for TN.

Allowing for variability in grading systems among studies, our results are similar to previously published results [11, 16]. The incidence of early pain relief was high (80.5 %) in our series. Pain relief was noted an average of 1.6 months after treatment. This time frame is consistent with previously published data [11, 16]. At our institution, we have therefore taken a similar approach to others by discussing further treatment options in which no degree of pain relief has been achieved in 6 months.

Our series is one of few to include only longer term follow-up of patients undergoing GKS for TN. An increasing body of literature suggests that the beneficial effect of stereotactic radiation on TN diminishes with time. At minimum follow-up of 3 years, 67 % were pain free (BNI I) and 75 % had good treatment outcome. At a mean last follow-up of 69 months, 32 % were free from any pain and 63 % were free from severe pain. Our results are strikingly similar to previously reported long-term analysis of TN pain outcomes. In a small series with minimum follow-up of 3 years, Park et al. [13] reported 17 % pain-free outcomes and 65 % with good pain outcome. Sheehan et al. [9] presented a subgroup of 39 patients (minimum 3-year follow-up) in which 70 % reported pain relief and 34 % reported an “ideal” outcome (BNI grade I). At a minimum follow-up of 36 months and an average follow-up of 48 months, Riesenburger et al. [11] reported on 53 patients with 58.5 % of patients experiencing good treatment outcome and 32.1 % of patients with pain-free outcome. In a more recently published series, Lee et al. [15] reported 27.3 % BNI I and II outcomes and 68 % good outcomes when data from 40 patients with minimum of 60-months follow-up and an average follow-up of 92.2 months were analyzed. Finally, in one of the largest series, Little and associates [12] reported actuarial data on 136 patients with a minimum follow-up duration of 4 years. At a mean follow-up of 6.3 years, they report 32 % of their patients being pain free off and off medication at 7 years and 83 % with good treatment outcomes, although they did include retreatment of patients [12]. In our series, we had lower response rates than those of Little et al. but results of retreatment were not included, which we would have expected to have increased the rate of overall successful pain relief.

Our incidence of new-onset facial numbness at 11 % was in accordance with previously published incidences. The reported incidence of facial numbness has ranged from 6 to 37 % [2, 5, 7, 9, 11, 16–18] with most series reporting new-onset numbness in ~10 % of treated patients. Similar to Riesenburger et al. [11], our series did not reveal any statistically significant correlation between known prognosticators such as age, gender, side of pain, duration of pain, or new-onset facial numbness and development of treatment failure at last follow-up.

However, a statistically significant correlation was found between age and recurrence of any pain with age >70 predicting a more favorable outcome radiosurgery. In their series examining long-term pain outcomes of TN, Han et al. [14] reported a similar correlation between older age >70 and favorable outcome in terms of pain recurrence after radiosurgery. The issue of age has been controversial in the literature as other series have reported a prognostic association between younger age and achieving and maintaining pain relief [8, 19]. Both of these studies, however, have been based on shorter follow-up period after radiosurgery. A correlation between history of prior ablative surgery and pain outcomes has long been established for TN pain recurrence after radiosurgery [1]. Given the low number of events in our series with only four patients having had undergone previous ablative procedures, that correlation could not be tested in our current study. Whether that could have biased our data in favor of a positive correlation between older age and pain recurrence after radiosurgery remains an uncertain question that needs to be addressed in larger sample sized studies.

Finally, although the median peripheral dose of 45 Gy that our institution has adopted is higher than the currently recommended dose (40 Gy) necessary to provide pain relief while avoiding toxicity [19, 20], we did not find a correlation between dose and either development of treatment failure at last follow-up or development of new bothersome facial numbness. Our analysis could have been underpowered in detecting a dose–outcome correlation, especially since the rate of trigeminal nerve dysfunction has been reported to decline with the increased follow-up duration [20]. Variations in other technical nuances of radiosurgery between institutions such as volume of nerve treated and anatomical location of the target (and hence the dose to the intraaxial portion of fifth nerve fibers in the brainstem) could have also confounded the impact of “high dose” (90 Gy) on both the probability of long-term pain control and fifth nerve dysfunction [21].

## Conclusion

GKRS is a safe, effective, and minimally invasive treatment modality for patients with medically intractable TN or those who are ineligible or refuse open surgery. Our results demonstrate that a single GKS treatment is associated with good outcomes in nearly 60 % of patients in an average follow-up period of approximately 6 years. While most pain recurrence develops within 6 months of treatment, treatment failure may still manifest years later. Our results also suggest that patients >70 presenting with medically intractable TN are good candidates for radiosurgery. The small sample size and retrospective nature of our study design limit the power of our outcome observations. Additionally, although pain intensity and numbness scales are validated tools for the quantification of pain and numbness, they are subjective outcome measures because they are dependent on personal interpretations and variation. The results of this study should, however, help clinicians provide important information to patients so they can have realistic expectations and be able to weigh the risks and benefits relative to the various available treatment options.

## References

[1] Yen CP, Schlesinger D, Sheehan JP (2011). Gamma Knife(R) radiosurgery for trigeminal neuralgia. Expert Rev Med Devices.

[2] Kondziolka D, Lunsford LD, Flickinger JC, Young RF, Vermeulen S, Duma CM (1996). Stereotactic radiosurgery for trigeminal neuralgia: a multiinstitutional study using the gamma unit. J Neurosurg.

[3] Brisman R, Khandji AG, Mooij RB (2002). Trigeminal nerve–blood vessel relationship as revealed by high-resolution magnetic resonance imaging and its effect on pain relief after gamma knife radiosurgery for trigeminal neuralgia. Neurosurgery.

[4] Han PP, Shetter AG, Smith KA, Fiedler JA, Rogers CL, Speiser B (1999). Gamma knife radiosurgery for trigeminal neuralgia: experience at the Barrow Neurological Institute. Stereotact Funct Neurosurg.

[5] Kondziolka D, Lunsford LD, Flickinger JC (2002). Stereotactic radiosurgery for the treatment of trigeminal neuralgia. Clin J Pain.

[6] McNatt SA, Yu C, Giannotta SL, Zee CS, Apuzzo ML, Petrovich Z (2005). Gamma knife radiosurgery for trigeminal neuralgia. Neurosurgery.

[7] Regis J, Metellus P, Hayashi M, Roussel P, Donnet A, Bille-Turc F (2006). Prospective controlled trial of gamma knife surgery for essential trigeminal neuralgia. J Neurosurg.

[8] Pollock BE, Phuong LK, Gorman DA, Foote RL, Stafford SL (2002). Stereotactic radiosurgery for idiopathic trigeminal neuralgia. J Neurosurg.

[9] Sheehan J, Pan HC, Stroila M, Steiner L (2005). Gamma knife surgery for trigeminal neuralgia: outcomes and prognostic factors. J Neurosurg.

[10] Urgosik D, Liscak R, Novotny J, Vymazal J, Vladyka V (2005). Treatment of essential trigeminal neuralgia with gamma knife surgery. J Neurosurg.

[11] Riesenburger RI, Hwang SW, Schirmer CM, Zerris V, Wu JK, Mahn K (2010). Outcomes following single-treatment Gamma Knife surgery for trigeminal neuralgia with a minimum 3-year follow-up. J Neurosurg.

[12] Little AS, Shetter AG, Shetter ME, Bay C, Rogers CL (2008). Long-term pain response and quality of life in patients with typical trigeminal neuralgia treated with gamma knife stereotactic radiosurgery. Neurosurgery.

[13] Park KJ, Kondziolka D, Kano H, Berkowitz O, Ahmed SF, Liu X (2012). Outcomes of gamma knife surgery for trigeminal neuralgia secondary to vertebrobasilar ectasia. J Neurosurg.

[14] Han JH, Kim DG, Chung HT, Paek SH, Kim YH, Kim CY (2009). Long-term outcome of gamma knife radiosurgery for treatment of typical trigeminal neuralgia. Int J Radiat Oncol Biol Phys.

[15] Lee JK, Choi HJ, Ko HC, Choi SK, Lim YJ (2012). Long term outcomes of gamma knife radiosurgery for typical trigeminal neuralgia-minimum 5-year follow-up. J Korean Neurosurg Soc.

[16] Maesawa S, Salame C, Flickinger JC, Pirris S, Kondziolka D, Lunsford LD (2001). Clinical outcomes after stereotactic radiosurgery for idiopathic trigeminal neuralgia. J Neurosurg.

[17] Fountas KN, Lee GP, Smith JR (2006). Outcome of patients undergoing gamma knife stereotactic radiosurgery for medically refractory idiopathic trigeminal neuralgia: Medical College of Georgia's experience. Stereotact Funct Neurosurg.

[18] Longhi M, Rizzo P, Nicolato A, Foroni R, Reggio M, Gerosa M (2007). Gamma knife radiosurgery for trigeminal neuralgia: results and potentially predictive parameters—part I: idiopathic trigeminal neuralgia. Neurosurgery.

[19] Flickinger JC, Pollock BE, Kondziolka D, Phuong LK, Foote RL, Stafford SL (2001). Does increased nerve length within the treatment volume improve trigeminal neuralgia radiosurgery? A prospective double-blind, randomized study. Int J Radiat Oncol Biol Phys.

[20] Pollock BE, Phuong LK, Foote RL, Stafford SL, Gorman DA (2001). High-dose trigeminal neuralgia radiosurgery associated with increased risk of trigeminal nerve dysfunction. Neurosurgery.

[21] Regis J, Tuleasca C (2011) Fifteen years of Gamma Knife surgery for trigeminal neuralgia in the Journal of Neurosurgery: history of a revolution in functional neurosurgery. J Neurosurg 115 Suppl:2–7 22401808

